# DEK is required for homologous recombination repair of DNA breaks

**DOI:** 10.1038/srep44662

**Published:** 2017-03-20

**Authors:** Eric A. Smith, Boris Gole, Nicholas A. Willis, Rebeca Soria, Linda M. Starnes, Eric F. Krumpelbeck, Anil G. Jegga, Abdullah M. Ali, Haihong Guo, Amom R. Meetei, Paul R. Andreassen, Ferdinand Kappes, Lisa M. Privette Vinnedge, Jeremy A. Daniel, Ralph Scully, Lisa Wiesmüller, Susanne I. Wells

**Affiliations:** 1 Division of Oncology; Cincinnati Children’s Hospital Medical Center, Cincinnati, OH, 45229, USA; 2 Department of Obstetrics and Gynecology; Ulm University, Ulm, 89075, Germany; 3 Department of Medicine, Division of Hematology-Oncology and Cancer Research Institute, Beth Israel Deaconess Medical Center and Harvard Medical School, Boston, MA, 02215, USA; 4 Chromatin Structure and Function Group, The Novo Nordisk Foundation Center for Protein Research, University of Copenhagen, Copenhagen, 2200, Denmark; 5 Division of Experimental Hematology and Cancer Biology; Cincinnati Children’s Hospital Medical Center, Cincinnati, OH, 45229, USA; 6 Institute of Biochemistry and Molecular Biology; Medical School, RWTH Aachen University, Aachen, 52074, Germany

## Abstract

DEK is a highly conserved chromatin-bound protein whose upregulation across cancer types correlates with genotoxic therapy resistance. Loss of DEK induces genome instability and sensitizes cells to DNA double strand breaks (DSBs), suggesting defects in DNA repair. While these DEK-deficiency phenotypes were thought to arise from a moderate attenuation of non-homologous end joining (NHEJ) repair, the role of DEK in DNA repair remains incompletely understood. We present new evidence demonstrating the observed decrease in NHEJ is insufficient to impact immunoglobulin class switching in DEK knockout mice. Furthermore, DEK knockout cells were sensitive to apoptosis with NHEJ inhibition. Thus, we hypothesized DEK plays additional roles in homologous recombination (HR). Using episomal and integrated reporters, we demonstrate that HR repair of conventional DSBs is severely compromised in DEK-deficient cells. To define responsible mechanisms, we tested the role of DEK in the HR repair cascade. DEK-deficient cells were impaired for γH2AX phosphorylation and attenuated for RAD51 filament formation. Additionally, DEK formed a complex with RAD51, but not BRCA1, suggesting a potential role regarding RAD51 filament formation, stability, or function. These findings define DEK as an important and multifunctional mediator of HR, and establish a synthetic lethal relationship between DEK loss and NHEJ inhibition.

The DNA-binding and chromatin-regulating *DEK* oncogene is expressed across multicellular eukaryotes and is highly conserved in mammals. Strong sequence conservation of the ψSAP-SAP and C-terminal DNA binding domains, as well as the lack of any known *DEK* paralogs, suggest stringent evolutionary pressure on this gene^1^,^2^. However, despite extensive biochemical, cellular, and clinical investigations into the DEK protein, molecular functions that explain this selective pressure remain poorly understood, as does the frequent over-expression of DEK in human cancers^3^,^4^,^5^,^6^,^7^,^8^.

In cultured cells, DEK has functions in chromatin remodeling^9^,^10^,^11^, DNA replication^9^,^12^, and mRNA splicing^13^. Depending on the experimental system chosen, DEK loss attenuates distinct oncogenic phenotypes such as proliferation^14^,^15^,^16^, survival^17^,^18^, and chemoresistance^14^,^17^,^19^. *Dek* knockout mice are viable and resistant to macroscopic tumor development^8^,^20^. Biochemically, there are no known enzymatic functions associated with DEK, but the protein self-multimerizes, induces positive supercoils in DNA through the ψ-SAP-SAP and C-terminal DNA binding domains, and preferentially binds cruciform DNA structures^21^,^22^,^23^,^24^. DEK also has roles in maintaining chromatin architecture^9^,^11^ and interacts with histones^10^,^25^ and chromatin modifiers^11^. Since the discovery of the *DEK* gene as a DEK-NUP214 fusion protein in AML^26^ and the discovery of elevated DEK expression in breast^3^,^27^, colorectal^5^,^28^, lung^4^,^29^, and several other types of cancer^1^, many systems have been used to investigate the pathological consequences of DEK over-expression. A prominent phenotype in cell models is the requirement of DEK for chemotherapy and radiation resistance. For example, expression of the DEK C-terminal domain in ataxia-telangiectasia fibroblasts partially restored radiation resistance, and the loss of DEK conferred sensitivity to DNA damaging agents in multiple cell types^14^,^19^,^30^. Mechanistically, our prior report found that DEK was required for optimal kinase activity of DNA-PK. This kinase is a key mediator of canonical non-homologous end joining (NHEJ), which repairs DNA double strand breaks (DSBs)^31^, and DEK loss correspondingly suppressed NHEJ^19^.

The observed NHEJ defects in DEK-deficient cells are unlikely to fully account for the severe sensitivity to genotoxic agents, especially DNA interstrand cross linkers and topoisomerase inhibitors^14^,^32^. This suggests additional roles for DEK in genotoxic drug tolerance and DNA repair. A common mechanism by which genotoxic agents induce cell death is through perturbation of replication fork progression^33^. A recent study determined that DEK attenuates DNA replication stress^12^ in a manner similar to RAD51 and FANCD2, factors well known for their function in both homologous recombination (HR) DSB repair and activities at arrested replication forks^34^,^35^,^36^,^37^.

HR requires the presence of a homologous template, often the sister-chromatid, to be used for repair, and is therefore largely confined to S/G2 phases of the cell cycle. By copying a homologous DNA sequence, HR is considered an error-free repair process that preserves genome integrity^38^. This signal cascade is initiated by the DSB sensor, ATM kinase. After localizing to a DSB, ATM autophosphorylates^39^,^40^, pATM catalyzes the phosphorylation of CHK2 to inhibit cell cycle progression^41^ and the H2AX histone to produce gamma-H2AX (γH2AX) epigenetic marks on both sides of the DSB. The γH2AX mark supports DSB repair by enhancing the recruitment of BRCA1 and key nucleases including the MRN complex and CtIP^38^,^42^,^43^,^44^. These factors coordinate DNA end processing into single strand DNA (ssDNA) 3′ tails^38^,^42^,^43^. The resulting ssDNA is initially coated by RPA, which is then efficiently replaced with a RAD51 filament through the combined activities of BRCA1, BRCA2, the RAD51 paralogs, and other factors^38^,^42^. This RAD51 filament catalyzes strand invasion, complementary strand annealing, and the formation of a stable synaptic complex with a homologous sequence on the sister chromatid^45^.

While the cellular regulation and choice between DSB-repair pathways remains incompletely understood^46^,^47^,^48^,^49^,^50^, NHEJ and HR factors have been shown to be mutually antagonistic as one pathway tends to compensate when the other is compromised^42^,^51^. For example, loss of critical HR gene functions in Fanconi Anemia mutant cells results in enhanced dependence upon NHEJ factors^52^,^53^,^54^ while HR repair efficiency is increased following the disruption of essential NHEJ factors by chemical inhibition or mutation of DNA-PK^51^,^55^,^56^. Compromising both the NHEJ and HR repair pathways may be synthetic lethal to cancer cells.

In this report, we demonstrate that DEK is required for the repair of DSBs by HR. Investigation of potential DEK functions in the HR pathway revealed that the protein was important for γH2AX activation, promoted the co-recruitment of BRCA1 and RAD51 to resected ssDNA, and formed a complex with RAD51 in a BRCA1-independent manner. The dependence of HR on DEK expression was underscored by the synthetic lethal relationship between DEK loss and NHEJ inhibition. Together, these results reveal a novel, multifunctional role for DEK in HR.

## Results

### DEK loss causes apoptosis in conjunction with DNA-PK inhibitors

**Figure 1:**
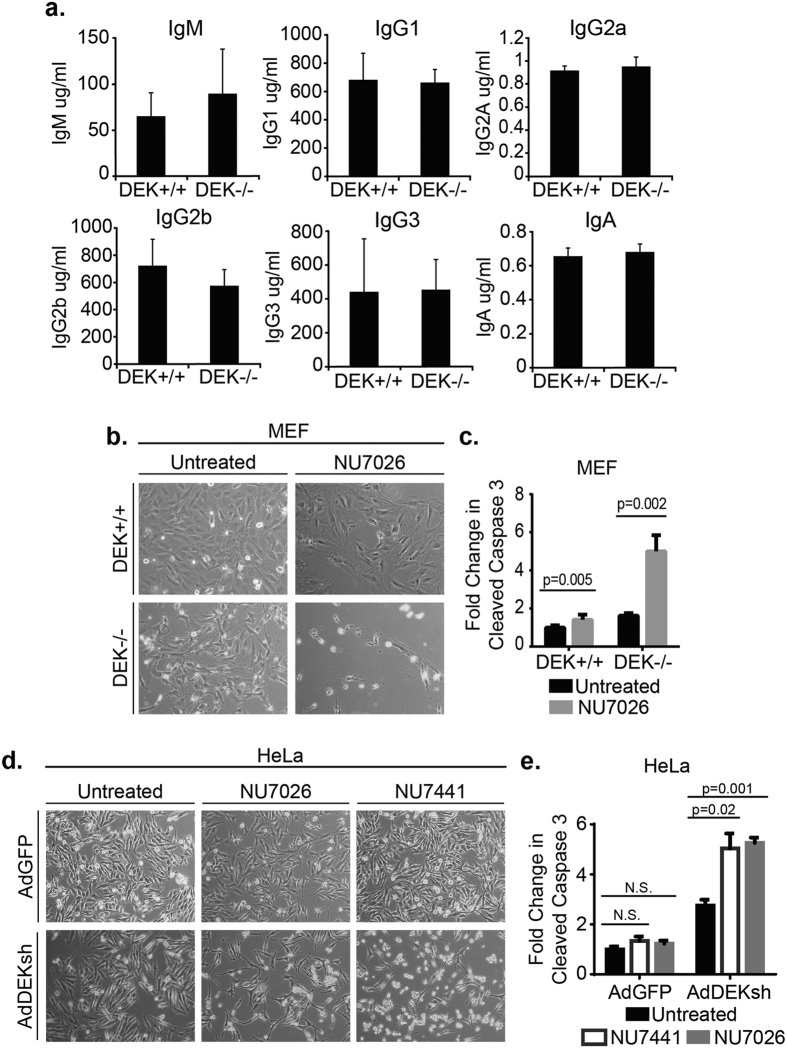
DEK loss causes apoptosis in conjunction with DNA-PK inhibitors. (**a**) Mouse serum immunoglobulin levels were quantified by ELISA (n = 3). (**b**) Representative live-cell images of mouse embryonic fibroblasts (MEFs) 72 hrs post treatment with a DNA-PK inhibitor (10x magnification). (**c**) DEK−/− MEF cells display increased apoptosis following 48 hr treatment with NU7026. (paired t-test, n = 6, mean ± SEM) (**d**) Hela cells were pre-treated with adenovirus for 24 hr prior to treatment with two different DNA-PK inhibitors. Representative images collected after incubating with drug for 48 hrs. (**e**) Both drugs demonstrated a significant increase in apoptosis at this time point. (paired t-test, n = 3, mean ± SEM).

We have previously shown that DEK knockout (DEK−/−) mouse embryonic fibroblasts (MEFs) harbor decreased NHEJ activity^19^. To determine the contribution of DEK to NHEJ activity *in vivo*, we measured the concentrations of immunoglobulins in DEK−/− mouse serum. Class switch recombination (CSR) from IgM to the IgA and IgG classes of immunoglobulins is an NHEJ-dependent process^31^, but DEK−/− mice were fully competent in producing all classes of immunoglobulins (Fig. 1a). This data suggests the residual NHEJ activity in the knockout mice is sufficient for CSR under physiologically normal *in vivo* conditions, despite the sensitivity of DEK-deficient cells to DNA damaging agents^14^,^19^. To determine if DEK-deficient cells require NHEJ for survival, we examined the need for NHEJ by inhibiting the upstream kinase, DNA-PK. We utilized two well-established DNA-PK inhibitors, NU7026 and NU7441, to prevent DNA-PKcs autophosphorylation and activation^57^,^58^,^59^,^60^. DEK−/− MEFs treated with the DNA-PK inhibitor NU7026 displayed dramatically more cell death than wild-type (DEK+/+) or untreated samples (Fig. 1b). Analysis of cleaved caspase 3 positive cells by flow cytometry revealed that the inhibitors were sufficient to induce a significant and specific 2–3 fold increase in apoptosis in the DEK−/− cells in the absence of exogenous DNA damaging agents (Fig. 1c). Similar experiments in HeLa cells infected with a well-characterized DEK knockdown versus control adenovirus vector (AdDEKsh versus AdGFP)^18^ revealed a similar 2-fold increase in apoptosis after treatment with either DNA-PK inhibitor, NU7026 or NU7441 (Fig. 1d,e). These data suggest that DEK-deficient cells, despite their attenuated NHEJ activity^19^, rely significantly on the remaining NHEJ for survival.

### DEK is required for HR DSB repair

**Figure 2:**
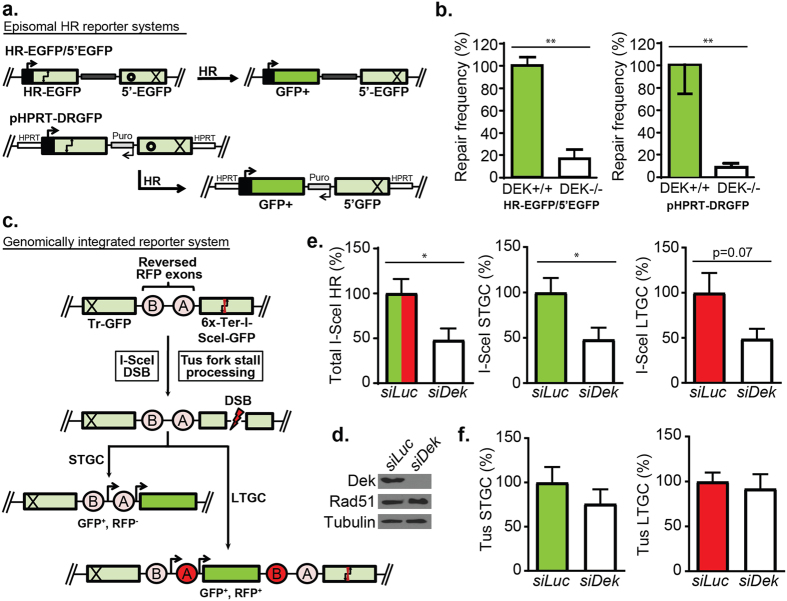
DEK is required for HR DSB repair. (**a**) Schematic of two episomal HR reporter plasmids. After co-transfection with an I-SceI plasmid, the mutant GFP gene (light green) site indicated by double arrows is cleaved. The circle represents the corresponding region of homology to the I-SceI site, and the X represents gene truncations. Expression of functional GFP (dark green) is dependent on HR repair of the cleaved plasmid. (**b**) GFP^+^ FACs quantification of repair frequency of the constructs in (A). (Mann-Whitney test, n = 12 for HR-EGFP/5′EGFP and n = 5 for pHPRT-DRGFP, mean ± SEM) (**c**) Schematic of the 6x*Ter* vector. Tus-Ter binding induces replication fork stalling at the 6x*Ter* array (red hourglass) while I-SceI creates a DSB at the location indicated by the double arrows. HR repair triggered by I-SceI or Tus expression results in short tract gene conversion (STGC, GFP^+^ RFP^−^), or long-tract gene conversion (LTGC) through the duplication and proper alignment of two synthetic *RFP* exons allowing RFP expression by alternative mRNA splicing (GFP^+^ RFP^+^). (**d**) Western blot analysis of 6x*Ter* mES cells after *siDek* treatment. (**e**) Quantification of total HR repair (STGC + LTGC) and individual STGC and LTGC repair FACs following I-SceI transfection. (Unpaired Welch’s t test, n = 6, mean ± SEM) (**f**) STGC and LTGC HR repair following Tus transfection. (Unpaired Welch’s t test, n = 6, mean ± SEM).

Several reports have described an antagonistic relationship between HR and NHEJ mechanisms of DSB repair^46^,^55^,^61^, and it is generally thought that loss of one pathway will result in compensation by the remaining repair modality^53^,^55^,^56^. Given the dependence of DEK-deficient cells on NHEJ and their inherent sensitivity to chemotherapeutics, we hypothesized that HR may be compromised in DEK−/− cells. To test this we first utilized two established episomal HR reporter systems^56^,^62^. These vectors harbor two defunct EGFP genes, the first bearing an I-SceI meganuclease cleavage site and the second bearing a truncated gene sequence (Fig. 2a). Co-transfection with an I-SceI expression vector induces a DSB that, when repaired by HR, generates a functional EGFP gene. Both the HR-EGFP/5′EGFP and pHPRT-DR-GFP reporters demonstrated a remarkable and severe loss of HR efficiency in DEK−/− MEFs (Fig. 2b). To compare the effects of DEK loss on chromosomal HR repair following direct or replication-dependent DSB induction, we utilized the recently published 6x*Ter*-HR reporter technology^63^. The 11CO/47 mouse embryonic stem (mES) cell line bearing a single copy of the 6x*Ter*-I-SceI-*GFP* reporter cassette integrated at the *Rosa26* locus quantifies HR either at an I-SceI endonuclease-induced DSB or at an adjacent Tus/Ter-induced stalled replication fork. Fork stalling is induced by Tus protein binding to an array of six *Ter* elements, forming a physical barrier that impedes approaching replication fork progression^63^ (Fig. 2c). Using this system, we quantified the impact of DEK depletion on error-free short tract gene conversion (STGC), producing GFP^+^RFP^−^ cells, and error-prone long tract gene conversion (LTGC), producing GFP^+^RFP^+^ cells through duplication and thus proper alignment of two synthetic exons of *RFP*. After co-transfecting *Dek* siRNA (Fig. 2D) and I-SceI, we found that *siDek* treated cells were significantly compromised for both total HR and STGC, and LTGC followed the same trend (Fig. 2e). This decrease was similar in magnitude to loss of the BRCA proteins^64^, but not as severe as RAD51 deficiency^63^. However, there was no significant decrease in STGC, LTGC, or total HR with Tus-induced DNA replication fork stalling (Fig. 2f). This suggests that DEK, unlike most HR factors^63^, is dispensable for replication fork associated repair but required for efficient repair of DSBs.

### DEK is necessary for the activation of γH2AX after ionizing radiation

**Figure 3:**
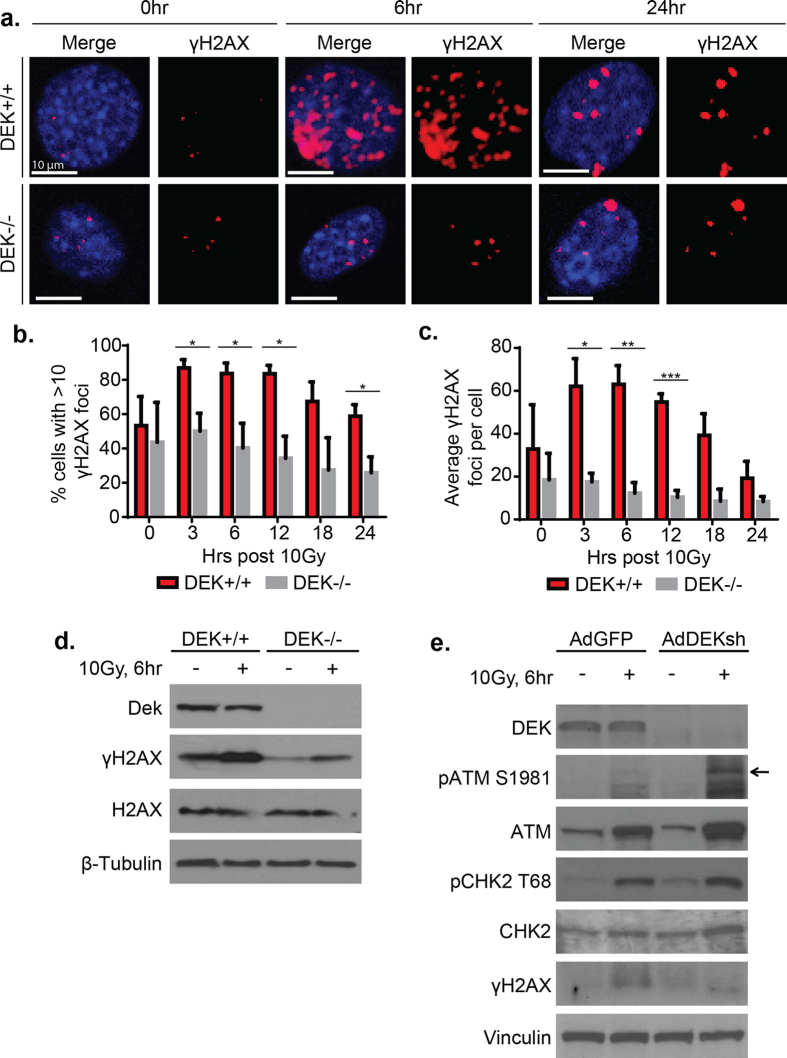
DEK is necessary for the activation of γH2AX after ionizing radiation. (**a**) MEFs were treated with 10 Gy of gamma ionizing radiation (IR) and stained for γH2AX. Representative cross sections from z-stack confocal images are shown. (**b**) Quantification of cells with >10 γH2AX foci in (A). At least 50 cells per time point were counted in each biological replicate, and all foci within the z-stack were quantified. *DEK*−/− MEFs were unable to activate γH2AX above baseline after irradiation. (paired t-test, n = 3, mean ± SEM). (**c**) Quantification of the average total number of γH2AX foci in each cell from (A). (paired t-test, n = 3, mean ± SEM) The number of foci per cell remained unchanged in *DEK*−/− MEFs after irradiation. (**d**) Western blot analysis of γH2AX in MEFs at 6 hs following irradiation with 10 Gy. (**e**) Western blot analysis of C33A human cancer cells infected with adenovirus 48 hr prior to receiving 10 Gy IR (see also Supplementary Fig. S1).

To understand how DEK functions in HR, we examined the consequences of DEK loss at successive steps in the repair pathway following irradiation (IR) mediated DSB induction. For these studies we used DEK−/− MEFs and AdDEKsh-infected HeLa cell systems, whose cell cycle progression was reported comparable to that of their respective DEK-proficient controls^18^,^19^. Beginning with the relevant upstream DNA damage sensor kinase, we found that ATM was strongly autophosphorylated in AdDEKsh-treated HeLa cells (Supplementary Fig. S1a) and functional as indicated by phosphorylation of pCHK2 T68, an ATM substrate (Supplementary Fig. S1b). Surprisingly, γH2AX phosphorylation was not enhanced in the AdDEKsh HeLa cells despite robustly activated pATM (Supplementary Fig. S1a). This held true and was even more severe in the DEK−/− MEF system where DEK was required to increase γH2AX phosphorylation above baseline by immunofluorescence (IF) (Fig. 3a). Both the total number of cells harboring γH2AX foci and the average number of foci per cell were severely attenuated from 3–24 hours after irradiation (Fig. 3b,c). These results were also confirmed by western blot analysis in MEFs and in DEK knockdown C33A cancer cells (Fig. 3d,e), suggesting that DEK is an important general contributor to γH2AX phosphorylation in IR-treated cells.

### Loading of RAD51 onto RPA-protected DNA is significantly reduced

**Figure 4:**
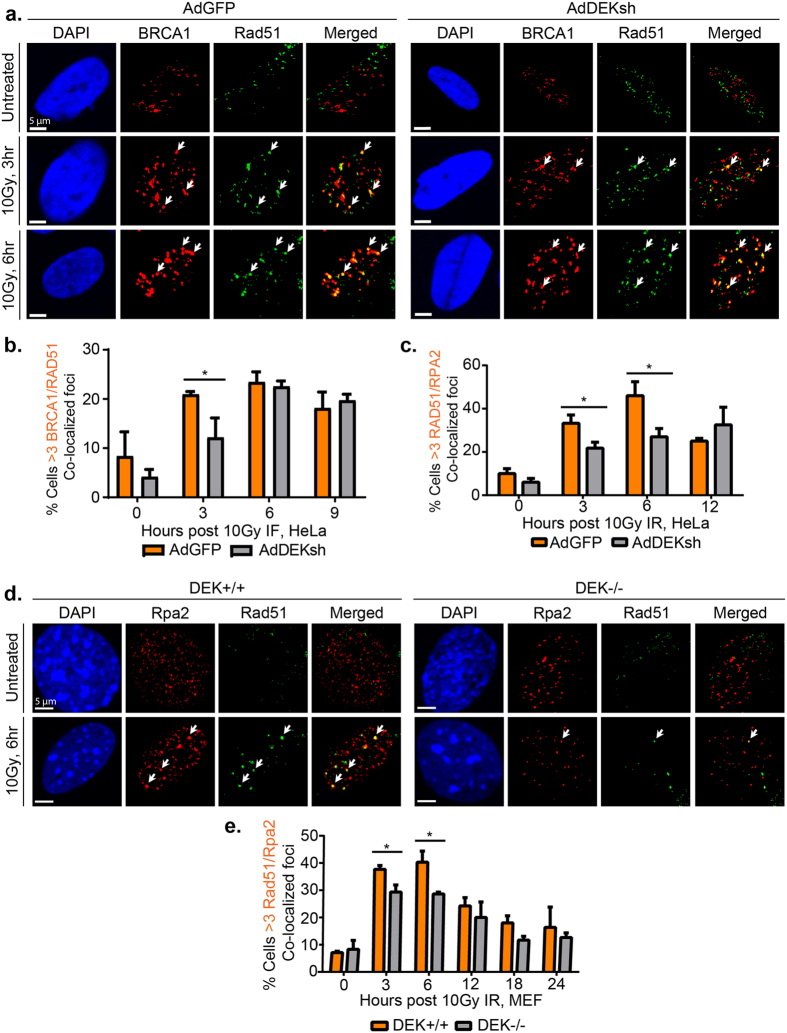
Loading of RAD51 onto RPA-protected DNA is significantly reduced. (**a**) HeLa cells were infected with 10 IU of adenovirus 48 hr prior to receiving 10 Gy and collection at 3, 6, and 12 hr post irradiation. Cells were subjected to immunofluorescence for BRCA1 (red) and RAD51 (far red, pseudo-colored green). Shown are representative images from untreated, 3 and 6hrs post irradiation. (**b**) Quantification of the co-localized foci from (A). Cells with ≥ 3 overlapping BRCA1 and RAD51 foci were counted as positive, and >100 cells were counted in three biological replicate experiments. (student’s t-test, n = 3, mean ± SEM, see also Supplementary Fig. S3). (**c**) HeLa cells were treated, collected, stained for RPA2 and RAD51 and quantified as in (A) and (B). (student’s t-test, n = 4, mean ± SEM, see also Supplementary Fig. S4). (**d**) To examine time points beyond 12 hrs, MEF cells were treated with 10 Gy, harvested at 3, 6, 12, 18, and 24 hr post treatment, and subjected to immunofluorescence for RPA2 (red) and RAD51 (far red, pseudo-colored green). Shown are representative images from untreated and 6 hr after irradiation. (**e**) Quantification of the co-localized foci from (D) performed as in (C).

DSB end-processing occurs downstream of the pATM-γH2AX signal amplification feedback loop and generates ssDNA overhangs that are bound and protected by RPA. End processing is coordinated by several factors, including the MRN complex and BRCA1^43^,^44^. Chromatin fractionation of HeLa cells revealed that chromatin recruitment of MRE11 and NBS1, components of the MRN complex, as well as BRCA1 were not affected by DEK status following IR (Supplementary Fig. S2a,b). Following end-processing, BRCA1 both co-localizes with and is necessary for robust RAD51 IF foci development^43^. Through assessing the quality and quantity of these foci, we determined whether DEK loss disrupts RAD51 loading onto RPA-bound ssDNA^43^. Since our BRCA1 antibody does not detect murine BRCA1, we performed these experiments in HeLa cells. DEK knockdown in HeLa cells had a mild, but statistically significant delay in BRCA1-RAD51 foci formation at 3 hours following IR (Fig. 4a,b) with no difference in individual BRCA1 or RAD51 foci formation at any time point (Supplementary Fig. S3). By 6 hours the co-localization of BRCA1 and RAD51 foci was indistinguishable from control cells. To determine if the delay in RAD51-BRCA1 co-localization correlated with impaired RAD51 loading onto RPA-protected ssDNA, we also determined the kinetics of RAD51 localization to RPA foci. In line with the delay with BRCA1-RAD51 co-localization, we found a significant decrease of RAD51-RPA2 co-localized foci at 3 and 6 hours post irradiation (Fig. 4c). A 24-hour time course in DEK+/+ and DEK−/− MEFs revealed that RAD51 loading was similarly attenuated (Fig. 4d,e). There was no difference in RAD51 foci quantity or quality (Supplementary Fig. S4a), but RPA2 foci were generally smaller in DEK−/− cells. DEK−/− cells also retained persistent RPA2 foci at later time points, suggesting a DNA repair defect (Supplementary Fig. S4b). In summary, DEK-deficient cells have a small reduction in the recruitment of RAD51 to BRCA1 foci, which results in a mild attenuation of RAD51 loading on RPA-coated ssDNA.

### DEK interacts with RAD51

**Figure 5:**
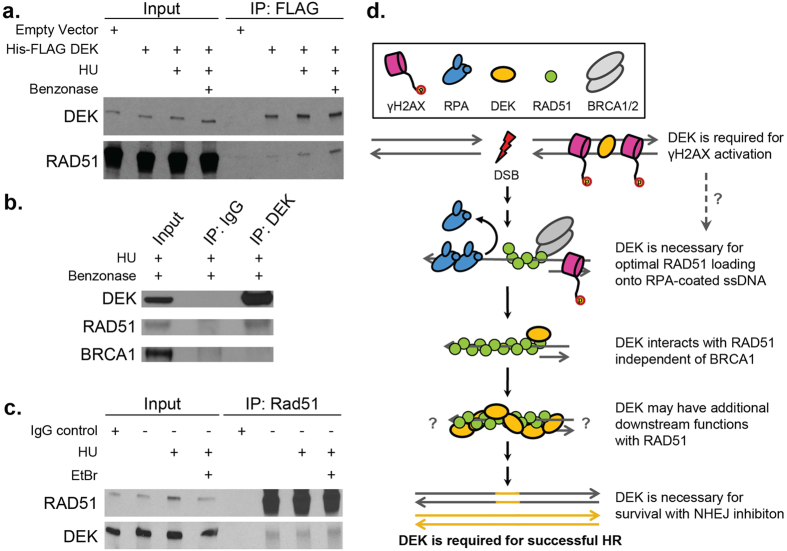
DEK interacts with RAD51 and is essential for multiple functions in HR DSB repair. (**a**) A FLAG IP performed in HeLa cells expressing His-FLAG tagged DEK, both in untreated and samples treated with 1 mM HU for 17 hrs. This identified RAD51 as a DNA-independent interacting factor. (**b**) RAD51 also co-immunoprecipitated with DEK in untransduced parental HeLa cells. (**c**) Reverse pulldown of RAD51 in parental HeLa cells confirmed DEK as an interacting partner. (**d**) DEK is required for successful HR of DSBs and functions at multiple levels in the repair cascade.

While RAD51 loading was not dramatically affected by DEK loss, physical DEK interactions with the RAD51 recombinase were possible. To test this, pMIEG His-FLAG-DEK expressing HeLa cells were left untreated or received 1 mM hydroxyurea (HU) for 15hrs to induce replication fork stalling and a low level of DSB generation as published previously^65^, prior to FLAG immunoprecipitation (IP). Under these conditions, we found that RAD51 complex formation occurred with His-FLAG DEK (Fig. 5a). Validation of the complex was performed by IP of endogenous DEK and reverse IP of RAD51 in parental HeLa cells (Fig. 5b,c). No BRCA1 interaction with DEK was observed (Fig. 5b), suggesting that DEK forms a separate complex from the standard BRCA-RAD51 homologue apparatus. The DEK-RAD51 complex was also observed in untreated and irradiated cells. Thus, DEK interacts with the HR recombinase RAD51 in the presence or absence of exogenous DNA damage (Supplementary Fig. S5).

## Discussion

Herein we demonstrate that DEK is required for the repair of DSBs by HR, and regulates multiple steps in the HR cascade by promoting γH2AX activation, enabling robust RAD51 loading, and forming a complex with RAD51 (Fig. 5d). This importance of DEK in HR was further underscored by the exquisite sensitivity of DEK-deficient cells to NHEJ loss through DNA-PK inhibition. In summary, this is the first report to describe DEK as a HR factor and to identify a synthetic lethal relationship that exists between DNA-PK inhibition and DEK loss.

According to the 6x*Ter* reporter assays (Fig. 2c–f), DEK appears to be required for HR only in the context of conventional DSBs, wherein DEK-deficient cells had a similar HR deficiency to BRCA1 loss^63^, but not in the context of stalled replication forks. However, this selectivity differs when DEK activities are compared to those of BRCA1, BRCA2, and RAD51, factors which are required for the HR repair triggered by both I-SceI induced DSBs and Tus-stalled replication forks^63^. Based on these observations, it is likely that DEK is expendable for replication-associated HR repair, but this by no means precludes non-HR functions at stalled replication forks. Indeed, published DNA fiber assays have elegantly shown that DEK can promote stalled replication fork restart and reduce γH2AX response to DNA replication stress^12^.

Differential γH2AX responses to irradiation (Fig. 3) versus chemotherapy-induced damage^12^,^19^ lends further support to a model where DEK is specifically required for HR repair of DSBs. Previous studies have found that loss of DEK enhances γH2AX activation following treatment with replication fork stalling agents^12^,^19^. While similar pATM activation was observed in response to stalling or irradiation, γH2AX phosphorylation was specifically not engaged after IR exposure. This suggests that DEK is specifically required for ATM-H2AX signal transduction under conditions of DSBs. While it is known that γH2AX is not strictly required for HR, its loss can slow the kinetics of the pathway and lead to a moderate decrease in repair efficiency^44^,^66^. Thus, the attenuation of BRCA1-RAD51 and RAD51-RPA2 foci co-localization (Fig. 4) is likely a consequence of failed γH2AX activation. These pulldown results, in which DEK interacted with RAD51 in a BRCA1-free complex (Fig. 5), further support this hypothesis as they suggest DEK is not part of the BRCA1-Palb2-BRCA2-RAD51 complex that facilitates RAD51 loading. However, we cannot presently rule out weak interactions or a role for BRCA in promoting the DEK-RAD51 interaction. Also, since these interactions were identified and confirmed in cervical cancer cells, it is uncertain if the DEK-RAD51 complex is a component of normal cell biology or specific to cancer cells.

Taken together these data support a model in which DEK has two distinct roles in the repair of DSBs by HR. First, DEK has an important function in γH2AX activation by ATM, but this is unlikely to be sufficient for the degree of HR deficiency observed in DEK-deficient cells^44^,^66^. Therefore, DEK must have a second function involving the DEK-RAD51 complex. With regard to a possible mechanism of action, DEK might play an early role in homologous recombination by supporting the initial formation of the RAD51-DNA filament. However, since BRCA1 and BRCA2 play key roles in this step and, since BRCA1 was not detectable in the DEK-RAD51 complex (Fig. 5b), we do not favor this scenario. Based on previous structural and cell-free studies, DEK binding to DNA stimulates self-multimerization and potential interactions with other proteins^22^,^67^,^68^. Furthermore, DEK preferentially binds cruciform over conventional linear DNA structures^24^, and harbors three putative DNA binding motifs^67^,^68^. Thus, one possible scenario is that DEK interacts with both the invading RAD51 filament and the sister chromatid to stabilize resulting D-loop structures. Such activities can now be readily explored through established biochemical assays^69^.

Since this is the first report to establish a role for DEK in HR, our work opens up multiple avenues for future study. At the molecular level, mechanisms whereby DEK promotes γH2AX activation specifically at DSBs and the nature and function of the complex with RAD51 remain unclear. Secondly, in the absence of known DEK homologues, detailed studies of the evolutionary history and origin of DEK are lacking despite its strong conservation across mammals and presence in plants. Interestingly, DEK is absent in bacteria and yeast, wherein mechanisms of HR are well described. It is possible that the early multicellular eukaryotes required a novel chromatin regulator to stabilize their increasingly complex genomes during DNA repair. Considering its role in chromatin modification and ATM-mediated γH2AX activation, its affinity for RAD51 and DNA structures similar to Holliday junctions, and its ability to stabilize DNA breaks for *in vitro* DNA ligation, DEK is a likely candidate to fill this niche^1^,^24^.

Still, further investigations into the activities of DEK in HR and NHEJ repair will remain important for deepening our knowledge of the evident evolutionary pressure on DEK and HR, understanding the multiple activities of DEK in HR, and for the development of small molecule inhibitors to target relevant functions of this oncogene. In support of these future therapeutic efforts, we identified a synthetic lethal relationship between DEK loss and canonical NHEJ inhibition, as well as a cellular phenotype that can be assessed for DEK inhibition *in vivo*. This is important as DEK is largely dispensable for cell division, and DEK−/− mice are healthy and viable^12^,^18^,^20^, suggesting a high therapeutic index for a potential anti-DEK drug. Thus, it is conceivable that DEK overexpressing tumors may regress without a need for adjuvant radiation or traditional chemotherapy if substituted for tumor-specific co-targeting of DEK and DNA-PK.

## Materials and Methods

### Cell culture, adenoviral infections, and viral transductions

HeLa and C33A cells were grown in Dulbecco’s Modified Essential Medium (DMEM) supplemented with 10% fetal bovine serum (FBS) and antibiotics. MEFs, generated previously^19^, were cultured in DMEM with 10% heat inactivated FBS, 100 μM MEM non-essential amino acids, 0.055 mM β-mercaptoethanol (BME), 2 mM L-glutamine, and 10 μg/ml gentamycin. Mouse embryonic stem (ES) cells were grown in ES media on fibroblast or gelatin substrate as previously described^63^. To induce DNA damage, cells were treated with γ-IR from a ^137^Cs source or 1 mM hydroxyurea (HU). Both 40 μM NU7026 and 2 μM NU7441 (Tocris, Bristol, UK) were used to specifically inhibit DNA-PK as previously published^59^,^70^. DEK knockdown was accomplished using the adenoviral AdDEKsh as compared to control AdGFP vectors at 10 infectious units (IU) per cell for 48 hr prior to IR treatment, as described previously^18^. The pMIEG His-FLAG-DEK retroviral vector was described recently^71^, and cells were transduced with virus for 24 hrs prior to sorting for GFP positive cells on a BD-FACSAria II flow cytometer.

### Serum Immunoglobulin ELISA Assays

Dek+/+ and Dek−/− littermate mice were born from heterozygous crosses on a mixed C57Bl6/S129 background as previously described^20^. Usage and handling of mice was performed with the approval of the Cincinnati Children’s Institutional Animal Care and Use Committee and complied with institutional, state, and federal guidelines and regulations as well as AAALAC accreditation standards. All mice were housed in specific pathogen free housing with ad libitum access to food and water. Blood was obtained by cardiac puncture of three DEK+/+ and three DEK−/− mice. To measure immunoglobulin (Ig) in the blood serum by ELISA, plates were coated with the following Southern Biotechnology Associates antibodies (Birmingham, AL, USA): anti-mouse IgM (no. 406501), IgA (no.556969), or IgG (no. 1030-01), and Ig was detected with horseradish peroxidase (HRP)-conjugated goat anti-mouse IgG1 (no. 1070-05), IgG3 (no. 1100-05), IgG2a (no.1080-05), IgG2b (no. 1090-05), IgA (no. 1040-05), or IgM (no. 1020-05). In all cases, wells were developed with the Ultra TMB peroxidase substrate system (Thermo Scientific) and OD was measured at 450 nm using a Fluostar Omega microplate reader (BMG-Labtech, Ortenberg, Germany).

### Cleaved caspase 3 flow cytometry

Cells were trypsinized, fixed in 4% PFA for 10 min at 37 °C, permeabilized in 90% ice-cold methanol for 30 min, and incubated with the cleaved caspase 3 cell signaling antibody for 1 hr. Analysis was performed on a BD FACSCanto II instrument.

### DNA repair reporter assays

MEFs were co-transfected with 2 μg of HR-EGFP/5′EGFP, pHPRT-DR-GFP, or an EGFP expressing plasmid (transfection control) and 2 μg pCMV-I-SceI using Fugene HD transfection reagent (Roche, Penzberg, Germany) prior to a 48 hr incubation^62^. Cells were collected and analyzed for EGFP expression by flow cytometry. Analysis of chromosomal HR repair was conducted using mouse ES cells bearing a single copy knock-in of the 6x*Ter*-HR reporter cassette targeted to the *Rosa26* locus^63^. For the 6x*Ter*-HR reporter cells, 1.6 × 10^5^ cells were cotransfected in suspension with 0.35 μg empty vector, pcDNA3β-myc NLS-Tus, or pcDNA3β-myc NLS-I-SceI, and 20 pmol ONTargetPlus-smartpool, (Dharmacon, Lafayette, CO), si*Luc* (p-002099-01-20), or si*Dek* (M-050260-00-0005) as described previously^63^. Transfected cells were analyzed by flow cytometry 72 hrs post transfection using a Becton Dickinson LSRII. GFP^+^ frequencies were measured in duplicate samples, 3–6 × 10^5^ total events were scored. Repair frequencies presented are corrected for background events and for transfection efficiency (60–85%). Transfection efficiency was measured by parallel transfection with 0.05 μg wild type *GFP* expression vector, 0.30 μg control vector and 20 pmol siRNA.

### Western blot analysis

Cell lysates were generated by lysis in NETN buffer (50 mM Tris-HCl pH = 7.4, 250 mM NaCl, 5 mM EDTA, 0.1% NP-40, 10 mM NaF, 200 μM Na_2_VO_3_, 0.5 mM PMSF, and 1x protease inhibitor cocktail) on ice for 30 min. Non-soluble debris was precipitated by a 5 min 12000 rpm spin at 4 °C and discarded. Lysate was treated with 4x loading buffer (0.5 M Tris-HCl pH 6.8, 277 mM SDS, 40% glycerol, bromophenol blue, 4% 2-mercaptoethanol), ran on a 10% SDS-PAGE gel, and transferred onto a PVDF membrane for 1–2 hr at 500 mA. The following Santa Cruz (Dallas, TX, USA) antibodies were used: BRCA1 (sc-6954) and RAD51 (sc-8349 and in-house aliquot B32). The following Cell Signaling (Danvers, MA, USA) antibodies were used: ATM (2873), GAPDH (5174), Chk2 (2662), pChk2 T68 (2661), Chk1 (2345), and pChk1 S345 (2341). Other antibodies included DEK (610948 BD Bioscience, San Jose, CA, USA), DEK (16448-1-AP, Protein Tech, Rosemont, IL, USA), DEK (in-house K-877)^72^, DNA-PKcs (ab1832, Abcam, Cambridge, MA, USA), pDNA-PKcs S2056 (ab18192), pATM S1981 (AF1655, R&D Systems, Minneapolis, MN), γH2AX (05-636, Millipore, Darmstadt, Germany), MRE11 (GTX70212, genetex, San Antonio, TX, USA), and NBS1 (NB100-143SS, Novus Biologicals, Littleton, CO, USA).

### Immunofluorescence microscopy

1–5 × 10^5^ cells were plated onto poly-d-lysine treated coverslips and allowed to attach prior to adenovirus infection and/or drug treatment. To stain, coverslips were fixed in 4% paraformaldehyde, antigens retrieved with a 0.2% Triton x-100 PBS solution, and cells blocked with PBS plus 5% normal goat serum and 0.3% Triton X-100. Antibody dilutions are as follows: BRCA1 (sc-6954), γH2AX 1:1000 (05-3636, Millipore), RPA2 1:300 (ab2175), RAD51 1:500 (sc-8349), goat anti-mouse Alexa Fluor 568 1:500 (A-11004, ThermoFischer Scientific, Waltham, MA), and goat anti-rabbit Alexa Fluor 633 1:500 (A-21070). Nuclei were counterstained with DAPI ProLong Gold (P-36931, Life Technologies). RPA and RAD51 foci were captured on a Carl Zeiss Apotome instrument, and foci within 100 cells counted per time point per replicate. BRCA1, RAD51, and RPA foci counts were performed by eye due to background signal from the antibodies. High resolution images were imaged on an inverted Nikon A1R GaAsP confocal microscope. Whole cell γH2AX foci were also collected on the confocal microscope using z-stacks and were quantified using Imaris 6 (Bitplane AG, Zurich, Switzerland).

### Chromatin fractionation

Chromatin fractionation was performed as described previously^73^ according to the schematic in Fig. 3d. Briefly, HeLa cells were infected with AdGFP or AdDEKsh 48 hr prior to receiving 10 Gy of IR. Six hours following IR, cells were counted to ensure equal numbers of cells per sample. Cell lysis and nuclear isolation was performed in a wash buffer (10 mM PIPES pH = 7.0, 1 mM EGTA, 0.1 M NaCl, 0.3 M sucrose, 0.5 M NaF, 0.5 mM Na_3_VO_4_, and 1x protease inhibitor cocktail) plus 1% Triton X-100. Serial separation of the precipitated nuclear fraction was performed first by treatment with wash buffer +20 U DNAseI (AM2235, ThermoFischer Scientific), followed by a 5 min incubation with wash buffer +0.5 M (NH_4_)SO_4_ on the resulting pellet. The resulting fractions were treated with 4x loading buffer.

### Immunoprecipitation (IP)

Subconfluent cells were treated with or without 1 mM HU for 15hrs. Cells were homogenized in lysis buffer (10 mM HEPES, pH = 7.9, 1.5 mM MgCl_2_, 10 mM KCl, 0.5 mM PMSF, 0.5 mM DTT, 1 mM NaVO_3_, 10 mM NaF, and 1x Sigma P5955 protease inhibitor cocktail), and the nuclear pellet was lysed in a nuclear lysis buffer (20 mM HEPES pH = 7.9, 400 mM NaCl, 1% Triton X-100, 0.1% NP-40, 10% Glycerol, 0.5 mM PMSF, 0.5 mM DTT, 1 mM NaVO_3_, 10 mM NaF, and 1x protease inhibitor cocktail) on ice. After ultracentrifugation, pMIEG and pMIEG His-FLAG-DEK supernatants were loaded onto anti-FLAG M2 affinity gel (A2200, Sigma, St. Louis, MO, USA). Parental cell supernatants were pretreated with antibodies for 1 hour prior to loading onto Protein A sepharose beads. For both IP experiments, lysates were treated with either 50 μg/ml ethidium bromide (EtBr) or 125 U of benzonase (E8263, Sigma) plus 2 mM MgCl_2_ as controls. After incubating overnight at 4 °C, the beads were washed, and the IP products collected by boiling in 2x Laemmli Buffer (12.5 mM Tris-HCl, pH = 6.8, 20% Glycerol, 4% SDS, 0.004% Bromophenol Blue, 10% 2-β-mercaptoethanol).

### Statistical Methods

Statistics were performed using Graphpad Prism 6 software (Graphpad Software Inc., San Diego, CA). Significance is indicated by asterisks (*p < 0.05, **p < 0.01, ***p < 0.001, ****p < 0.0001), and the number of independent biological replicates and type of analysis is indicated in the Fig. legends.

## Additional Information

**How to cite this article:** Smith, E. A. *et al*. DEK is required for homologous recombination repair of DNA breaks. *Sci. Rep.* **7**, 44662; doi: 10.1038/srep44662 (2017).

**Publisher's note:** Springer Nature remains neutral with regard to jurisdictional claims in published maps and institutional affiliations.

## Supplementary Material

Supplementary Information

## References

[b1] Privette Vinnedge L. M., Kappes F., Nassar N. & Wells S. I. Stacking the DEK: from chromatin topology to cancer stem cells. Cell Cycle 12, 51–66, doi: 10.4161/cc.23121 (2013). 23255114 PMC3570517

[b2] Waldmann T., Scholten I., Kappes F., Hu H. G. & Knippers R. The DEK protein–an abundant and ubiquitous constituent of mammalian chromatin. Gene 343, 1–9, doi: 10.1016/j.gene.2004.08.029 (2004). 15563827

[b3] Ying G. & Wu Y. DEK: A novel early screening and prognostic marker for breast cancer. Mol Med Rep 12, 7491–7495, doi: 10.3892/mmr.2015.4380 (2015). 26459608

[b4] Wang X. . High expression of oncoprotein DEK predicts poor prognosis of small cell lung cancer. Int J Clin Exp Pathol 7, 5016–5023 (2014). 25197373 PMC4152063

[b5] Lin L. . DEK over expression as an independent biomarker for poor prognosis in colorectal cancer. BMC cancer 13, 366, doi: 10.1186/1471-2407-13-366 (2013). 23902796 PMC3751154

[b6] Datta A. . Oncoprotein DEK as a tissue and urinary biomarker for bladder cancer. BMC cancer 11, 234, doi: 10.1186/1471-2407-11-234 (2011). 21663673 PMC3130704

[b7] Lin D. . Identification of DEK as a potential therapeutic target for neuroendocrine prostate cancer. Oncotarget 6, 1806–1820 (2015). 25544761 10.18632/oncotarget.2809 PMC4359333

[b8] Adams A. K. . DEK promotes HPV-positive and -negative head and neck cancer cell proliferation. Oncogene 34, 868–877, doi: 10.1038/onc.2014.15 (2015). 24608431 PMC4160430

[b9] Alexiadis V. . The protein encoded by the proto-oncogene DEK changes the topology of chromatin and reduces the efficiency of DNA replication in a chromatin-specific manner. Genes & development 14, 1308–1312 (2000). 10837023 PMC316669

[b10] Ko S. I. . Regulation of histone acetyltransferase activity of p300 and PCAF by proto-oncogene protein DEK. FEBS letters 580, 3217–3222, doi: 10.1016/j.febslet.2006.04.081 (2006). 16696975

[b11] Kappes F. . The DEK oncoprotein is a Su(var) that is essential to heterochromatin integrity. Genes & development 25, 673–678, doi: 10.1101/gad.2036411 (2011). 21460035 PMC3070930

[b12] Deutzmann A. . The human oncoprotein and chromatin architectural factor DEK counteracts DNA replication stress. Oncogene 34, 4270–4277, doi: 10.1038/onc.2014.346 (2015). 25347734

[b13] Soares L. M., Zanier K., Mackereth C., Sattler M. & Valcarcel J. Intron removal requires proofreading of U2AF/3′ splice site recognition by DEK. Science 312, 1961–1965, doi: 10.1126/science.1128659 (2006). 16809543

[b14] Khodadoust M. S. . Melanoma proliferation and chemoresistance controlled by the DEK oncogene. Cancer Res 69, 6405–6413, doi: 10.1158/0008-5472.CAN-09-1063 (2009). 19679545 PMC2727675

[b15] Privette Vinnedge L. M. . The human DEK oncogene stimulates beta-catenin signaling, invasion and mammosphere formation in breast cancer. Oncogene 30, 2741–2752, doi: 10.1038/onc.2011.2 (2011). 21317931 PMC3117026

[b16] Wise-Draper T. M. . The human DEK proto-oncogene is a senescence inhibitor and an upregulated target of high-risk human papillomavirus E7. J Virol 79, 14309–14317, doi: 10.1128/JVI.79.22.14309-14317.2005 (2005). 16254365 PMC1280217

[b17] Kappes F. . DEK is a poly(ADP-ribose) acceptor in apoptosis and mediates resistance to genotoxic stress. Mol Cell Biol 28, 3245–3257, doi: 10.1128/MCB.01921-07 (2008). 18332104 PMC2423161

[b18] Wise-Draper T. M. . Apoptosis inhibition by the human DEK oncoprotein involves interference with p53 functions. Mol Cell Biol 26, 7506–7519, doi: 10.1128/MCB.00430-06 (2006). 16894028 PMC1636856

[b19] Kavanaugh G. M. . The human DEK oncogene regulates DNA damage response signaling and repair. Nucleic acids research 39, 7465–7476, doi: 10.1093/nar/gkr454 (2011). 21653549 PMC3177200

[b20] Wise-Draper T. M. . Overexpression of the cellular DEK protein promotes epithelial transformation *in vitro* and *in vivo*. Cancer Res 69, 1792–1799, doi: 10.1158/0008-5472.CAN-08-2304 (2009). 19223548 PMC2650744

[b21] Waldmann T., Eckerich C., Baack M. & Gruss C. The ubiquitous chromatin protein DEK alters the structure of DNA by introducing positive supercoils. The Journal of biological chemistry 277, 24988–24994, doi: 10.1074/jbc.M204045200 (2002). 11997399

[b22] Kappes F., Scholten I., Richter N., Gruss C. & Waldmann T. Functional domains of the ubiquitous chromatin protein DEK. Mol Cell Biol 24, 6000–6010, doi: 10.1128/MCB.24.13.6000-6010.2004 (2004). 15199153 PMC480879

[b23] Bohm F. . The SAF-box domain of chromatin protein DEK. Nucleic acids research 33, 1101–1110, doi: 10.1093/nar/gki258 (2005). 15722484 PMC549417

[b24] Waldmann T., Baack M., Richter N. & Gruss C. Structure-specific binding of the proto-oncogene protein DEK to DNA. Nucleic acids research 31, 7003–7010 (2003). 14627833 10.1093/nar/gkg864 PMC290247

[b25] Sawatsubashi S. . A histone chaperone, DEK, transcriptionally coactivates a nuclear receptor. Genes & development 24, 159–170, doi: 10.1101/gad.1857410 (2010). 20040570 PMC2807351

[b26] von Lindern M. . The translocation (6;9), associated with a specific subtype of acute myeloid leukemia, results in the fusion of two genes, dek and can, and the expression of a chimeric, leukemia-specific dek-can mRNA. Mol Cell Biol 12, 1687–1697 (1992). 1549122 10.1128/mcb.12.4.1687-1697.1992 PMC369612

[b27] Liu S. . DEK overexpression is correlated with the clinical features of breast cancer. Pathology international 62, 176–181, doi: 10.1111/j.1440-1827.2011.02775.x (2012). 22360505

[b28] Martinez-Useros J. . DEK is a potential marker for aggressive phenotype and irinotecan-based therapy response in metastatic colorectal cancer. BMC cancer 14, 965, doi: 10.1186/1471-2407-14-965 (2014). 25515240 PMC4300837

[b29] Liu X. . Significance of DEK overexpression for the prognostic evaluation of non-small cell lung carcinoma. Oncology reports 35, 155–162, doi: 10.3892/or.2015.4365 (2016). 26530274

[b30] Meyn M. S., Lu-Kuo J. M. & Herzing L. B. Expression cloning of multiple human cDNAs that complement the phenotypic defects of ataxia-telangiectasia group D fibroblasts. American journal of human genetics 53, 1206–1216 (1993). 7504406 PMC1682482

[b31] Kotnis A., Du L., Liu C., Popov S. W. & Pan-Hammarstrom Q. Non-homologous end joining in class switch recombination: the beginning of the end. Philos Trans R Soc Lond B Biol Sci 364, 653–665, doi: 10.1098/rstb.2008.0196 (2009). 19008195 PMC2660918

[b32] Yamazaki H. . SiRNA knockdown of the DEK nuclear protein mRNA enhances apoptosis and chemosensitivity of canine transitional cell carcinoma cells. Vet J 204, 60–65, doi: 10.1016/j.tvjl.2015.02.009 (2015). 25773167

[b33] Cheung-Ong K., Giaever G. & Nislow C. DNA-damaging agents in cancer chemotherapy: serendipity and chemical biology. Chem Biol 20, 648–659, doi: 10.1016/j.chembiol.2013.04.007 (2013). 23706631

[b34] Hashimoto Y., Ray Chaudhuri A., Lopes M. & Costanzo V. Rad51 protects nascent DNA from Mre11-dependent degradation and promotes continuous DNA synthesis. Nature structural & molecular biology 17, 1305–1311, doi: 10.1038/nsmb.1927 (2010). PMC4306207 20935632

[b35] Schlacher K., Wu H. & Jasin M. A distinct replication fork protection pathway connects Fanconi anemia tumor suppressors to RAD51-BRCA1/2. Cancer cell 22, 106–116, doi: 10.1016/j.ccr.2012.05.015 (2012). 22789542 PMC3954744

[b36] Lossaint G. . FANCD2 binds MCM proteins and controls replisome function upon activation of s phase checkpoint signaling. Molecular cell 51, 678–690, doi: 10.1016/j.molcel.2013.07.023 (2013). 23993743

[b37] Gonzalez-Prieto R., Munoz-Cabello A. M., Cabello-Lobato M. J. & Prado F. Rad51 replication fork recruitment is required for DNA damage tolerance. EMBO J 32, 1307–1321, doi: 10.1038/emboj.2013.73 (2013). 23563117 PMC3642682

[b38] Krejci L., Altmannova V., Spirek M. & Zhao X. Homologous recombination and its regulation. Nucleic acids research 40, 5795–5818, doi: 10.1093/nar/gks270 (2012). 22467216 PMC3401455

[b39] Lee J. H. & Paull T. T. ATM activation by DNA double-strand breaks through the Mre11-Rad50-Nbs1 complex. Science 308, 551–554, doi: 10.1126/science.1108297 (2005). 15790808

[b40] Bakkenist C. J. & Kastan M. B. DNA damage activates ATM through intermolecular autophosphorylation and dimer dissociation. Nature 421, 499–506, doi: 10.1038/nature01368 (2003). 12556884

[b41] Zannini L., Delia D. & Buscemi G. CHK2 kinase in the DNA damage response and beyond. J Mol Cell Biol 6, 442–457, doi: 10.1093/jmcb/mju045 (2014). 25404613 PMC4296918

[b42] Ceccaldi R., Rondinelli B. & D’Andrea A. D. Repair Pathway Choices and Consequences at the Double-Strand Break. Trends Cell Biol 26, 52–64, doi: 10.1016/j.tcb.2015.07.009 (2016). 26437586 PMC4862604

[b43] Prakash R., Zhang Y., Feng W. & Jasin M. Homologous recombination and human health: the roles of BRCA1, BRCA2, and associated proteins. Cold Spring Harb Perspect Biol 7, a016600, doi: 10.1101/cshperspect.a016600 (2015). 25833843 PMC4382744

[b44] Scully R. & Xie A. Double strand break repair functions of histone H2AX. Mutation research 750, 5–14, doi: 10.1016/j.mrfmmm.2013.07.007 (2013). 23916969 PMC3818383

[b45] Jasin M. & Rothstein R. Repair of strand breaks by homologous recombination. Cold Spring Harb Perspect Biol 5, a012740, doi: 10.1101/cshperspect.a012740 (2013). 24097900 PMC3809576

[b46] Escribano-Diaz C. . A Cell Cycle-Dependent Regulatory Circuit Composed of 53BP1-RIF1 and BRCA1-CtIP Controls DNA Repair Pathway Choice. Molecular cell, doi: 10.1016/j.molcel.2013.01.001 (2013). 23333306

[b47] Yun M. H. & Hiom K. CtIP-BRCA1 modulates the choice of DNA double-strand-break repair pathway throughout the cell cycle. Nature 459, 460–463, doi: 10.1038/nature07955 (2009). 19357644 PMC2857324

[b48] Polato F. . CtIP-mediated resection is essential for viability and can operate independently of BRCA1. J Exp Med 211, 1027–1036, doi: 10.1084/jem.20131939 (2014). 24842372 PMC4042650

[b49] Xu G. . REV7 counteracts DNA double-strand break resection and affects PARP inhibition. Nature 521, 541–544, doi: 10.1038/nature14328 (2015). 25799992 PMC4671316

[b50] Onyango D. O., Howard S. M., Neherin K., Yanez D. A. & Stark J. M. Tetratricopeptide repeat factor XAB2 mediates the end resection step of homologous recombination. Nucleic acids research 44, 5702–5716, doi: 10.1093/nar/gkw275 (2016). 27084940 PMC4937314

[b51] Allen C., Halbrook J. & Nickoloff J. A. Interactive competition between homologous recombination and non-homologous end joining. Molecular cancer research: MCR 1, 913–920 (2003). 14573792

[b52] Kee Y. & D’Andrea A. D. Expanded roles of the Fanconi anemia pathway in preserving genomic stability. Genes & development 24, 1680–1694, doi: 10.1101/gad.1955310 (2010). 20713514 PMC2922498

[b53] Pace P. . Ku70 corrupts DNA repair in the absence of the Fanconi anemia pathway. Science 329, 219–223, doi: 10.1126/science.1192277 (2010). 20538911

[b54] Adamo A. . Preventing nonhomologous end joining suppresses DNA repair defects of Fanconi anemia. Molecular cell 39, 25–35, doi: 10.1016/j.molcel.2010.06.026 (2010). 20598602

[b55] Neal J. A. . Inhibition of homologous recombination by DNA-dependent protein kinase requires kinase activity, is titratable, and is modulated by autophosphorylation. Mol Cell Biol 31, 1719–1733, doi: 10.1128/MCB.01298-10 (2011). 21300785 PMC3126343

[b56] Pierce A. J., Hu P., Han M., Ellis N. & Jasin M. Ku DNA end-binding protein modulates homologous repair of double-strand breaks in mammalian cells. Genes & development 15, 3237–3242, doi: 10.1101/gad.946401 (2001). 11751629 PMC312854

[b57] Lombardi A. J. . Acquisition of Relative Interstrand Crosslinker Resistance and PARP Inhibitor Sensitivity in Fanconi Anemia Head and Neck Cancers. Clinical cancer research: an official journal of the American Association for Cancer Research 21, 1962–1972, doi: 10.1158/1078-0432.CCR-14-2616 (2015). 25609062 PMC4401632

[b58] Leahy J. J. . Identification of a highly potent and selective DNA-dependent protein kinase (DNA-PK) inhibitor (NU7441) by screening of chromenone libraries. Bioorg Med Chem Lett 14, 6083–6087, doi: 10.1016/j.bmcl.2004.09.060 (2004). 15546735

[b59] Veuger S. J., Curtin N. J., Richardson C. J., Smith G. C. & Durkacz B. W. Radiosensitization and DNA repair inhibition by the combined use of novel inhibitors of DNA-dependent protein kinase and poly(ADP-ribose) polymerase-1. Cancer Res 63, 6008–6015 (2003). 14522929

[b60] Callen E. . Essential role for DNA-PKcs in DNA double-strand break repair and apoptosis in ATM-deficient lymphocytes. Molecular cell 34, 285–297, doi: 10.1016/j.molcel.2009.04.025 (2009). 19450527 PMC2709792

[b61] Chapman J. R. . RIF1 Is Essential for 53BP1-Dependent Nonhomologous End Joining and Suppression of DNA Double-Strand Break Resection. Molecular cell, doi: 10.1016/j.molcel.2013.01.002 (2013). PMC3594748 23333305

[b62] Bohringer M. & Wiesmuller L. Fluorescence-based quantification of pathway-specific DNA double-strand break repair activities: a powerful method for the analysis of genome destabilizing mechanisms. Subcell Biochem 50, 297–306, doi: 10.1007/978-90-481-3471-7_15 (2010). 20012588

[b63] Willis N. A. . BRCA1 controls homologous recombination at Tus/Ter-stalled mammalian replication forks. Nature 510, 556–559, doi: 10.1038/nature13295 (2014). 24776801 PMC4118467

[b64] Chandramouly G. . BRCA1 and CtIP suppress long-tract gene conversion between sister chromatids. Nat Commun 4, 2404, doi: 10.1038/ncomms3404 (2013). 23994874 PMC3838905

[b65] Petermann E., Orta M. L., Issaeva N., Schultz N. & Helleday T. Hydroxyurea-stalled replication forks become progressively inactivated and require two different RAD51-mediated pathways for restart and repair. Molecular cell 37, 492–502, doi: 10.1016/j.molcel.2010.01.021 (2010). 20188668 PMC2958316

[b66] Yuan J., Adamski R. & Chen J. Focus on histone variant H2AX: to be or not to be. FEBS letters 584, 3717–3724, doi: 10.1016/j.febslet.2010.05.021 (2010). 20493860 PMC3695482

[b67] Devany M., Kotharu N. P. & Matsuo H. Solution NMR structure of the C-terminal domain of the human protein DEK. Protein science: a publication of the Protein Society 13, 2252–2259, doi: 10.1110/ps.04797104 (2004). 15238633 PMC2279821

[b68] Devany M., Kappes F., Chen K. M., Markovitz D. M. & Matsuo H. Solution NMR structure of the N-terminal domain of the human DEK protein. Protein science: a publication of the Protein Society 17, 205–215, doi: 10.1110/ps.073244108 (2008). 18227428 PMC2222715

[b69] Raynard S. & Sung P. Assay for human Rad51-mediated DNA displacement loop formation. Cold Spring Harb Protoc 2009, pdb prot5120, doi: 10.1101/pdb.prot5120 (2009). 20147015 PMC2956496

[b70] Ciszewski W. M., Tavecchio M., Dastych J. & Curtin N. J. DNA-PK inhibition by NU7441 sensitizes breast cancer cells to ionizing radiation and doxorubicin. Breast Cancer Res Treat 143, 47–55, doi: 10.1007/s10549-013-2785-6 (2014). 24292814

[b71] Matrka M. C. . DEK over-expression promotes mitotic defects and micronucleus formation. Cell Cycle 14, 3939–3953, doi: 10.1080/15384101.2015.1044177 (2015). 25945971 PMC4825741

[b72] Kappes F. . Phosphorylation by protein kinase CK2 changes the DNA binding properties of the human chromatin protein DEK. Mol Cell Biol 24, 6011–6020, doi: 10.1128/MCB.24.13.6011-6020.2004 (2004). 15199154 PMC480878

[b73] Wang X., Andreassen P. R. & D’Andrea A. D. Functional interaction of monoubiquitinated FANCD2 and BRCA2/FANCD1 in chromatin. Mol Cell Biol 24, 5850–5862, doi: 10.1128/MCB.24.13.5850-5862.2004 (2004). 15199141 PMC480901

