# Supplementary material for: DEK is required for homologous recombination repair of DNA breaks
Source: Sci Rep. 2017 Mar 20;7:44662. doi: 10.1038/srep44662 (PMC5357905; doi:10.1038/srep44662)
Supplement: Supplementary Information [file srep44662-s1.doc]

**DEK is required for homologous recombination repair of DNA breaks**

Eric A. Smith^1^, Boris Gole^2^, Nicholas A. Willis^3^, Rebeca Soria^4^, Linda M. Starnes^4^, Eric F. Krumpelbeck^1^, Anil G. Jegga^1^, Abdullah M. Ali^5^, Haihong Guo^6^, Amom R. Meetei^5^, Paul R. Andreassen^5^, Ferdinand Kappes^6^, Lisa M. Privette Vinnedge^1^, Jeremy A. Daniel^4^, Ralph Scully^3^, Lisa Wiesmüller^2^, Susanne I. Wells^1,*^

^1^Division of Oncology; Cincinnati Children’s Hospital Medical Center; Cincinnati, OH, 45229; USA. ^2^Department of Obstetrics and Gynecology; Ulm University; Ulm, 89075; Germany.

^3^Department of Medicine, Division of Hematology-Oncology and Cancer Research Institute, Beth Israel Deaconess Medical Center and Harvard Medical School, Boston, MA 02215.

^4^Chromatin Structure and Function Group, The Novo Nordisk Foundation Center for Protein Research, University of Copenhagen, Copenhagen 2200, Denmark.

^5^Division of Experimental Hematology and Cancer Biology; Cincinnati Children’s Hospital Medical Center; Cincinnati, OH, 45229; USA.

^6^Institute of Biochemistry and Molecular Biology; Medical School, RWTH Aachen University; Aachen, 52074; Germany.

*To whom correspondence should be addressed. Tel: 513-636-5986; Fax 513-636-3549; E-mail: [Susanne.Wells@cchmc.org](mailto:Susanne.Wells@cchmc.org);

Present Address: Dr. SI Wells, Division of Oncology, Cincinnati Children’s Hospital Medical Center, MLC 7013, 3333 Burnet Avenue, Cincinnati, OH 45229, USA.

**SUPPLEMENTAL DATA**


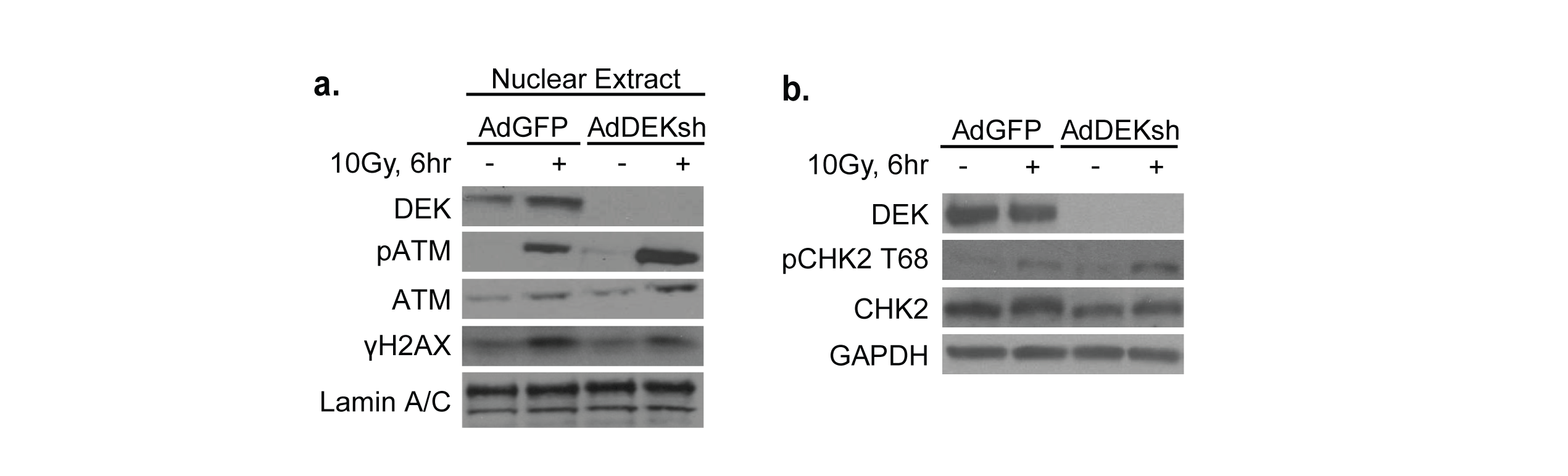


Fig. S1. DEK knockdown HeLa cells have increased active pATM, but no increase in γH2AX compared to control cells. **(a)** Nuclear fraction was performed on HeLa cells treated with adenovirus and 10 Gy IR (same experiment as in Supplementary Fig. S2). **(b)** ATM activation in (a) resulted in CHK2 phosphorylation in AdDEKsh treated HeLa cells after IR treatment. HeLa cells were treated with adenovirus 48 hr prior to treatment with 10 Gy IR. Whole cell extracts were collected in (b) and used for western blot analysis.


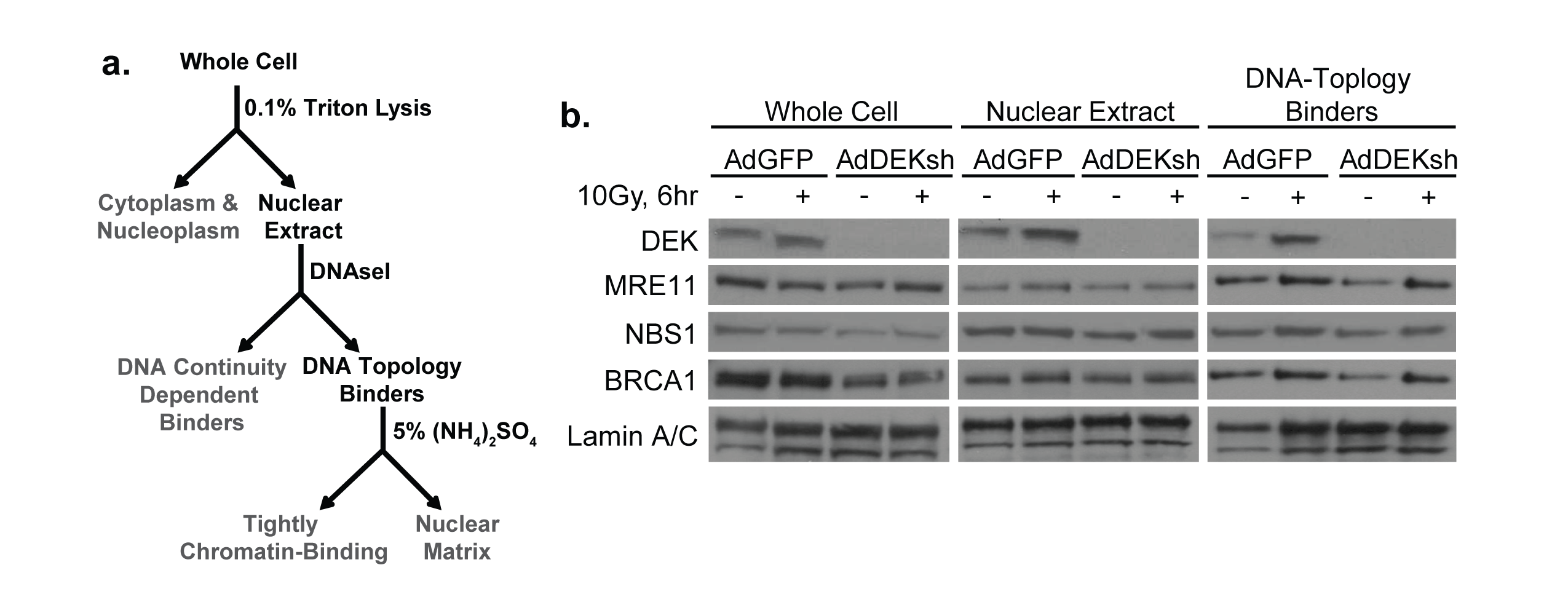


Fig. S2. DEK knockdown does not affect chromatin recruitment of the DSB end-processing complex. **(a)** Chromatin fractionation was performed on HeLa cells by serial centrifugation as outlined in the schematic. Whole cells were lysed with a 0.1% Triton X-100 solution, and the insoluble nuclear extract was further fractionated by stepwise DNAse I treatment and high salt conditions. (5% (NH_4_)_2_SO_4_ solution) **(b)** Chromatin fractionation was performed as outlined in (A) following a 48 hr incubation with adenovirus and 6 hr incubation post irradiation (n=2). The nuclear extract in Supplementary Fig. S1a is from the same experiment.


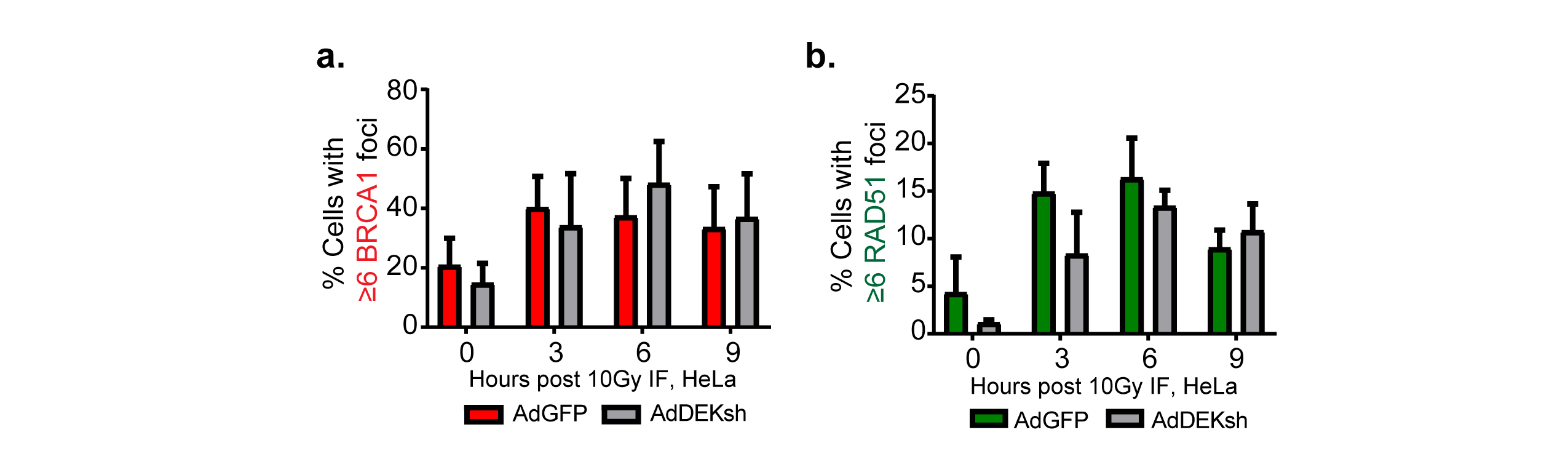


Fig. S3. DEK loss does not affect the total number of cells positive for BRCA1 foci. HeLa cells were infected with 10 IU of AdGFP or AdDEKsh 48 hr prior to receiving 10 Gy of ionizing radiation (IR). Cells were labelled for BRCA1 and Rad51, and cells with **(a)** ≥6 BRCA1 foci or **(b)** ≥6 RAD51 foci were counted as positive. (>100 cells per sample, unpaired t-test, n=3, mean ± SEM).


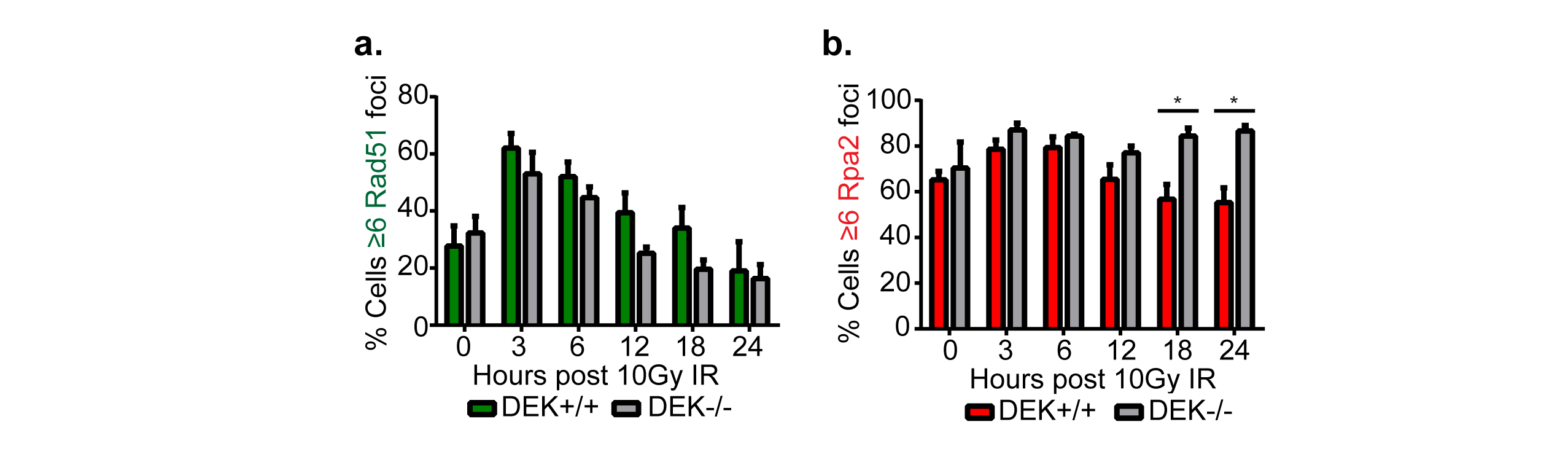


Fig. S4. DEK loss does not affect the total number of cells positive for RAD51 foci, but RPA foci positive cells remain elevated in *Dek* KO cells at 18 and 24 hrs post IR. **(a-b)** Quantification of RAD51 (green) and RPA (red) from the experiments in Figure 4B. Cells with ≥6 individual RPA (B) or RAD51 foci (C) were counted as positive, and 100 cells were counted in four biological replicate experiments (unpaired t-test, n=4, mean ± SEM).


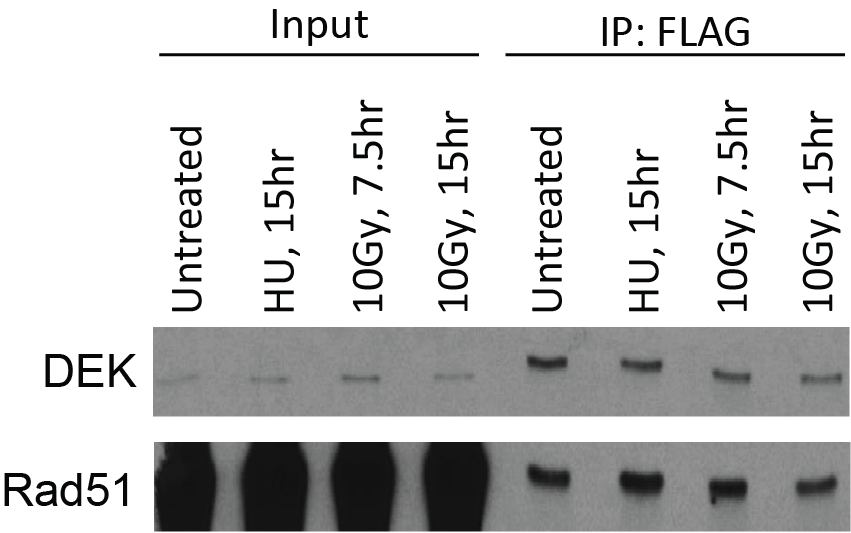


Fig. S5. DEK interacts with RAD51 in untreated cells and in cells that received 10Gy IR. Pulldown was performed MIEG-His-FLAG-DEK transduced HeLa cells as in Fig. 5a.
